# Ohio State Veterinary Medical Association

**Published:** 1901-02

**Authors:** 


### OHIO STATE VETERINARY MEDICAL ASSOCIATION.

FOR the first time in its history the State Association held its annual meeting at the State University. The Association was entertained by the College of Veterinary Medicine. Considering the fact that the veterinarians of the State were nearly all very busy for this time of the year, practice being good, the turn-out was a representative one. The first session was opened by Dr. W. O. Thompson, President of the University, who delivered an address of welcome which was enthusiastically received. Dr. Thompson dwelt upon the rapidly expanding field of veterinary medicine and the high place it must eventually assume as a profession. He announced the intention of the University to augment its present equipment and facilities by the erection of suitable buildings for the Veterinary College, which, when completed, would be second to none in the country. A regular programme followed. Dr. T. B. Cotton's paper on "Tuberculosis of the Horse" was followed by a very animated discussion. His assertion that "light-complexioned persons and sorrel horses are all more or less tuberculous" did not seem to find the applause of the professional gentlemen present, who shelled the doctor's trenches with lyddite bombs. An interesting address entitled "Notes from City Practice," by Dr. E. H. Shepard, of Cleveland, was extremely pithy and full of good thoughts. The doctor exhibited an interesting calculus, weighing sixteen ounces, which he had recently removed from the bladder of a mare, through the urethra, without the aid of a knife or "crusher."

A "smoker" added to the social feature of the evening session. Reports of committees were read, discussed, and approved. The question of parturient paresis of the ox and the value of Schmidt's treatment were gone into at some length. Some members reported no better results from its use than from former methods. However, the general opinion seemed to indicate that the mortality in this disease had been greatly reduced since the adoption of Schmidt's method.

The following morning a clinic was held at the College Hospital, where three hours were spent performing the more common surgical operations.

A new operating-table for dogs, designed by Dr. O. V. Brumley, of the

Ohio State University, was favorably commented upon. The new patho-anatomical laboratory was visited by all interested in that line of work.

After adopting resolutions of thanks to the University and Columbus veterinarians one of the best meeting ever held by the Association came to a close. D. S.

